# The Plasma Interleukin (IL)-35 Level and Frequency of Circulating IL-35^+^ Regulatory B Cells are Decreased in a Cohort of Chinese Patients with New-onset Systemic Lupus Erythematosus

**DOI:** 10.1038/s41598-019-49748-z

**Published:** 2019-09-13

**Authors:** Zhuang Ye, Yanfang Jiang, Dejun Sun, Wei Zhong, Ling Zhao, Zhenyu Jiang

**Affiliations:** 1 Department of Rheumatology, First Hospital, Jilin University, Changchun, 130021 China; 2 Genetic Diagnosis Center, First Hospital, Jilin University, Changchun, 130021 China; 3 Key Laboratory of Zoonosis Research, Ministry of Education, First Hospital, Jilin University, Changchun, 130021 China; 4 Department of Biomedicine, Institute for Regeneration Medicine, Jilin University, Changchun, 130021 China; 5 Department of Rheumatology, First Hospital of Qiqihaer City, Qiqihaer, 161006 China; 6 Department of Mathematics, Hong Kong University of Science and Technology, Clear Water Bay, Hong Kong, 999077 China

**Keywords:** Ankylosing spondylitis, Skeleton

## Abstract

Systemic lupus erythematosus (SLE) is a multisystemic autoimmune disease that is associated with the destruction of immune tolerance and activation of B cells. Interleukin (IL)-35 and IL-35-producing (IL-35^+^) regulatory B cells (Bregs) have been demonstrated to possess immunosuppressive functions, but their roles in the initiation and early development of SLE have not been explored. Here, we measured and compared the frequencies of blood regulatory B cell subsets and the concentrations of plasma IL-35, IL-10, IL-17A, tumor necrosis factor (TNF)-α, and interferon (IFN)-γ in 47 Chinese patients with newly diagnosed SLE and 20 matched healthy controls (HCs). The SLE patients had decreased percentages of IL-35^+^ B cells and IL-10^+^ B cells among the total blood B cells as well as decreased concentrations of plasma IL-35. In addition, higher levels of plasma IL-10, IFN-γ, TNF-α, and IL-17 along with higher frequencies of circulating plasma and memory B cells were observed in the SLE patients. The percentage of IL-35^+^ Bregs and the serum IL-35 level were inversely correlated with the SLE disease activity index and the erythrocyte sedimentation rate (ESR) levels. Our results indicate that IL-35^+^ Bregs and IL-35 may play protective roles in SLE initiation and progression.

## Introduction

Systemic lupus erythematosus (SLE) is a chronic inflammatory autoimmune disease featured with high amounts of autoantibody production and systemic clinical manifestations, including fatigue, joint pain, rash, and fever^1^. The precise etiopathogenesis of SLE remains unknown. However, destruction of immune tolerance and activation of B cells, through antibody-independent functions as well as antigen processing and presentation to T cells, have been identified as essential pathogenic factors in the evolution of lupus^2,3^. Therefore, the approach of targeting B cells for immune regulation has been considered to be therapeutically effective in the treatment of patients with SLE.

Recently, a B cell subset, namely regulatory B cells (Bregs), has been revealed to possess immunosuppressive functions and support immunological tolerance^4,5^. Bregs are able to secrete interleukin (IL)-10, IL-35, and transforming growth factor β (TGF-β) as well as inhibit the proliferation of pathogenic T cells and other proinflammatory lymphocytes^6^, thus potently suppressing immunopathogenesis. The well-illustrated mechanism underlying B cell immunoregulation is the production of IL-10^7^. For example, in autoimmune diseases including SLE and rheumatoid arthritis, IL-10-producing (IL-10^+^) Bregs have been shown to be functionally impaired and decreased in numbers^6,8,9^. However, other IL-10-independent mechanisms of immunosuppressive functions of Bregs, including the production of IL-35, IgM, or adenosine, have been proposed, but they still remain poorly understood.

Like IL-10, IL-35 is a cytokine produced by Bregs, but it belongs to the IL-12 family. IL-35 also has been demonstrated to possess immunosuppressive functions^10^. For example, IL-35 is reported to limit immune responses through inhibiting effector T cell functions^11^. In mice with experimental autoimmune uveitis, IL-35 has been revealed to induce IL-10 secretion of B cells and expand the number of IL-10^+^ Bregs to suppress autoimmune disease^12^. In a mouse model of experimental autoimmune encephalitis (EAE), the immunoregulatory function of IL-35 in B cells and the expansion of IL-35-producing (IL-35^+^) Bregs have been proven to be indispensable for mice to recover from EAE^13^. Therefore, IL-35 and IL-35^+^ Bregs have recently been confirmed to be important players in mediating immune tolerance in patients with autoimmune diseases. However, very few studies have investigated the association between the abundance of IL-35/IL-35^+^ Bregs and SLE progression, and the potential roles of IL-35/IL-35^+^ Bregs in the pathogenesis of SLE still need to be better elucidated. In addition, whether and how the abundance of IL-35/IL-35^+^ Bregs correlates with that of IL-10/IL-10^+^ Bregs have not been intensively explored, especially in Chinese patients with SLE.

In this study, we determined the frequencies of blood circulating IL-35^+^ Bregs, IL-10^+^ Bregs, and B cell subsets at different maturation statuses in 47 new-onset SLE patients and 20 healthy controls (HCs). In addition, we measured the concentrations of plasma cytokines, including IL-35, IL-10, IL-17, tumor necrosis factor (TNF)-α, and interferon (IFN)-γ, and further evaluated their correlations among the abundances of the Breg subsets, the concentrations of plasma cytokines, and other clinical indicators of SLE patients.

## Results

### Comparisons of demographic and clinical characteristics of SLE patients and HCs

As shown in Table 1, comparisons of the clinical information between 47 new-onset SLE patients and 20 matched HCs indicated no significant difference in terms of age, gender, or white blood cell count. Compared with the HCs, the patients with newly diagnosed SLE demonstrated significantly elevated levels of plasma immunoglobulin (Ig) G, IgA, IgM, erythrocyte sedimentation rate (ESR), and C-reactive protein (CRP), while they had decreased levels of complement factors C3 and C4. In addition, varied SLE Disease Activity Index (SLEDAI) scores (mean value, 18; range, 3–38) were identified among the SLE patients. Furthermore, over half of these SLE patients had positive sera for anti-double stranded (ds) DNA antibody (59.57%) and anti-Smith (Sm) antibody (53.19%) (Table 1).

**Table 1:** Clinical and laboratory characteristics of the subjects in this study.

Parameters	SLE patients	HCs
	(n = 47)	(n = 20)
Age (years, range)	27 (19–58)	29 (19–60)
Gender ratio (female/male)	41/6	17/3
Disease duration	47 (100%)^†^	ND
SLEDAI	18 (3–38)	ND
Positive anti-dsDNA	28 (59.57%)	ND
Positive anti-Sm	25 (53.19%)	ND
Positive antinuclear antibodies	43 (91.49)	ND
ESR (mm/h)	49 (6–120)*	7.8 (5–12)
CRP (mg/L)	6.75 (0.50–162)*	2 (0.50–3)
C3 (IU/mL)	0.43 (0.05–1.53)*	1.39 (1.1–1.5)
C4 (U/mL)	0.07 (0.00–0.35)*	0.27 (0.1–0.3)
IgG (g/L)	17.20 (4.38–36.70)*	11.00 (7.00–16.00)
IgA (g/L)	3.10 (0.25–6.62)*	2.30(0.70–4.00)
IgM (g/L)	17.20 (4.38–36.70)*	1.5 (0.40–2.30)
WBC (10^9^/L)	4.71 (1.08–17.70)	6.77 (3.50–9.50)

Note: Data are shown as the median (range) or number of cases. CRP, C-reactive protein; ESR, erythrocyte sedimentation rate; HCs, healthy controls; IgA, Immunoglobulin A; IgG, Immunoglobulin G; IgM, Immunoglobulin M; ND, undetectable; SLE, systemic lupus erythematosus; SLEDAI, SLE disease activity index; WBC, white blood cell counts.

Normal ranges of the following parameters: C3, 0.9–1.8 units/mL; C4, 0.1–0.4 units/mL; CRP, 0–3 mg/L; ESR, 0–15 mm/h; IgA, 0.7–4.0 g/L; IgG, 7.00–16.00 g/L; IgM, 0.4–2.3 g/L; WBC, 3.50–9.50 × 10^9^/L. *P < 0.05 versus HCs; ^†^, all the patients displayed clinical symptoms for less than three months.

### SLE patients exhibited decreased frequencies of circulating IL-35^+^ Bregs, IL-10^+^ Bregs, and IL-5^+^ Bregs among blood B cells

**Figure 1:**
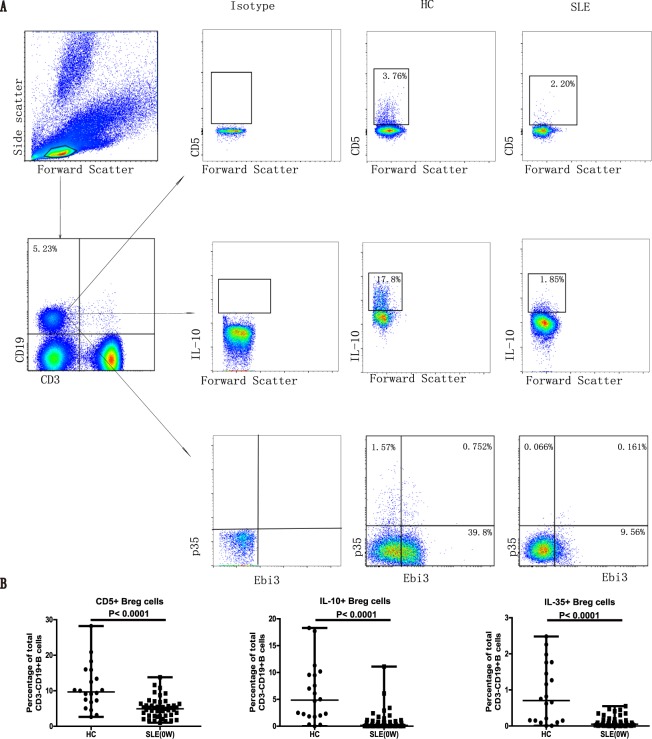
New-onset SLE patients had significantly lower percentages of CD5^+^ Bregs, IL-10^+^ Bregs, and IL-35^+^ Bregs among the peripheral blood circulating B cells. (**A**,**B**) PBMCs were isolated from the newly diagnosed SLE patients (n = 47, at baseline) and healthy controls (HCs, n = 20). The percentages of different B cell subsets, including CD5^+^ Bregs, IL-10^+^ Bregs, and IL-35^+^ Bregs, were analyzed by flow cytometry. (**A**) The representative flow cytometric profiles show the gating strategy and staining patterns of CD3^−^CD19^+^ total B cells and different subsets of Bregs in the SLE and HC groups. IL-12A (p35) and Epstein-Barr virus-induced 3 (EBI3) are the two subunits of IL-35. (**B**) Summarized data of the percentages of different subsets of Bregs among the total blood B cells. Each dot represents the percentage of the indicated Bregs from an individual subject. All *P* values < 0.05, as determined by the Mann-Whitney U test.

It has been demonstrated previously that Bregs can secrete IL-10 and IL-35 to suppress the overactivated immunity in autoimmune diseases^7^. To identify the potential role of IL-35^+^ B cells and other Breg subsets, we analyzed the percentages of circulating IL-35^+^ B cells, IL-10^+^ B cells, and CD5^+^ Bregs between newly diagnosed SLE patients and HCs by flow cytometry (Fig. 1A). The results indicated a significantly decreased frequency of IL-35^+^ B cells among the total CD3^−^CD19^+^ blood B cells in the SLE patients, compared with that in the HCs (Fig. 1B). Similarly, the SLE patients also demonstrated a significant reduction in the frequencies of IL-10^+^ Bregs and CD5^+^ Bregs in peripheral blood B lymphocytes (Fig. 1B).

### The plasma IL-35 level was decreased in new-onset SLE patients and was correlated with the percentages of IL-35^+^ Bregs and IL-10^+^ Bregs

**Figure 2:**
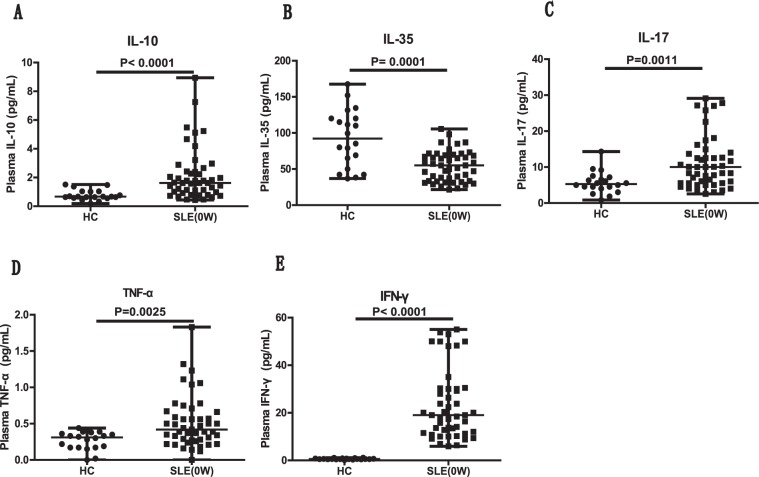
New-onset SLE patients had significantly increased levels of plasma IL-10, IL-17, IFN-γ, and TNF-α as well as decreased levels of plasma IL-35. (**A**–**E**) Plasma obtained from newly diagnosed SLE patients (n = 47, at baseline) and HCs (n = 20) was subjected to ELISA and CBA assays to quantitate the cytokine levels. The levels of plasma IL-10 (**A**), IL-35 (**B**), IL-17 (**C**), TNF-α (**D**), and IFN-γ (**E**) in the SLE and HC groups are summarized. Each dot represents the level of the indicated plasma cytokine from an individual subject. All *P* values < 0.05, as determined by the Mann-Whitney U test.

**Figure 3:**
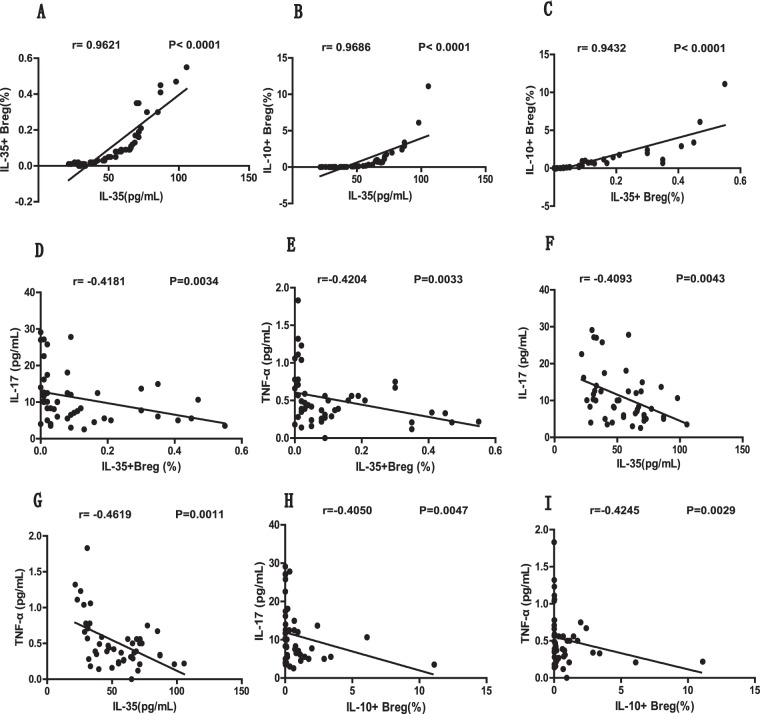
The analyses of correlations between the percentages of different Breg subsets and plasma cytokine levels in newly diagnosed SLE patients. (**A**,**B**) The correlation between the plasma IL-35 level and the percentage of circulating IL-35^+^ Bregs (**A**) or IL-10^+^ Bregs (**B**) among the total B cells in newly diagnosed SLE patients. (**C**) The correlation between the percentages of circulating IL-35^+^ Bregs and IL-10^+^ Bregs. (**D**,**E**) The correlation between the percentage of circulating IL-35^+^ Bregs and the level of plasma IL-17 (**D**) or TNF-α (**E**). (**F**,**G**) The correlation between the level of plasma IL-35 and the level of plasma IL-17 (**F**) or TNF-α (**G**). (**H**,**I**) The correlation between the percentage of circulating IL-10^+^ Bregs and the level of plasma IL-17 (**H**) or TNF-α (**I**). All P values < 0.05, as determined by the Spearman’s rank correlation test.

Next, we determined the concentrations of IL-35 as well as other effector and proinflammatory cytokines, including IL-10, IL-17, TNF-α, and IFN-γ, in the plasma of all subjects. Compared with the HCs, the patients with newly diagnosed SLE had notably decreased levels of plasma IL-35 (Fig. 2B) and significantly elevated levels of plasma IL-10 (Fig. 2A), IL-17 (Fig. 2C), TNF-α (Fig. 2D), and IFN-γ (Fig. 2E). It is worth noting that the plasma IL-35 level was positively correlated with the percentages of IL-35^+^ B cells (*P* < 0.0001, r = 0.9621; Fig. 3A) and IL-10^+^ B cells (*P* < 0.0001, r = 0.9686; Fig. 3B) in the new-onset SLE patients. Similarly, we also found a significant correlation between the frequencies of IL-35^+^ B cells and IL-10^+^ B cells (*P* < 0.0001, r = 0.9432; Fig. 3C). Moreover, the plasma concentration of IL-17 showed inverse correlations with the plasma IL-35 levels (*P* = 0.0043, r = −0.4093; Fig. 3F) as well as the percentages of IL-35^+^ B cells (*P* = 0.0034, r = −0.4181; Fig. 3D) and IL-10^+^ B cells (*P* = 0.0047, r = −0.4050; Fig. 3H). Inverse correlations between TNF-α and the plasma IL-35 level (*P* = 0.0011, r = −0.4619; Fig. 3G), the percentage of IL-35^+^ B cells (*P* = 0.0033, r = −0.4204; Fig. 3E), and the percentage of IL-10^+^ B cells (*P* = 0.0029, r = −0.4245; Fig. 3I) were also identified. However, we did not observe any correlations between the level of plasma IL-10 or IFN-γ and the frequencies of Breg subsets or plasma IL-35 levels in the SLE patients (data not shown).

### The plasma IL-35 level was negatively correlated with the frequency of circulating CD27^+^CD38^−^ memory B cells in new-onset SLE patients

**Figure 4:**
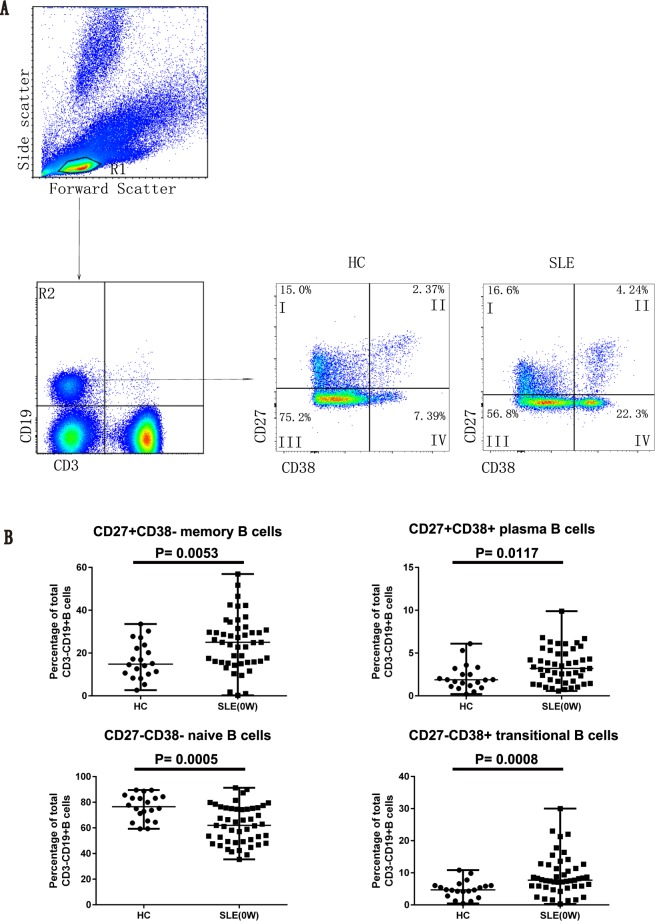
Assessing the percentages of memory, plasma, transitional, and naive B cell subsets among the circulating CD3^−^CD19^+^ B cells in SLE patients and HCs. (**A**,**B**) PBMCs collected from newly diagnosed SLE patients (n = 47, at baseline) and HCs (n = 20) were subjected to flow cytometry to quantitate the percentages of CD27^+^CD38^−^ memory B cells, CD27^+^CD38^+^ plasma B cells, CD27^−^CD38^+^ transitional B cells, and CD27^−^CD38^−^ naive B cells among the total CD3^−^CD19^+^ B cells. (**A**) The representative flow cytometric profiles show the gating strategy and staining patterns of total CD3^−^CD19^+^ B cells as well as different subsets of B cells in the SLE and HC groups. R1, lymphocytes; R2, CD3^−^CD19^+^ T cells; I, CD27^+^CD38^−^ memory B cells; II, CD27^+^CD38^+^ plasma B cells; III, CD27^−^CD38^+^ transitional B cells; IV, CD27^−^CD38^−^ naive B cells. (**B**) Summarized data of the percentages of different B cell subsets among the total blood B cells. Each dot represents the percentage of the indicated B cell subsets from an individual subject. All *P* values < 0.05, as determined by the Mann-Whitney U test.

**Figure 5:**
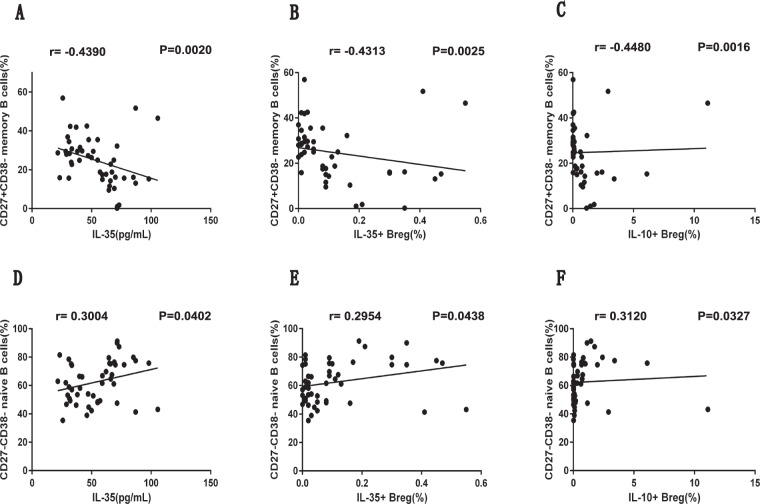
The analyses of correlations between the percentage of Breg subsets or plasma IL-35 level and the percentage of memory B cells or naive B cells in newly diagnosed SLE patients. (**A**–**C**) The correlation between the percentage of CD27^+^CD38^−^ memory B cells and the level of plasma IL-35 (**A**) or the percentage of IL-35^+^ (**B**) or IL-10^+^ (**C**) Breg subsets in the SLE patients. (**D**–**F**) The correlation between the percentage of CD27^−^CD38^−^ naive B cells and the level of plasma IL-35 (**D**) or the percentage of IL-35^+^ (**E**) or IL-10^+^ (**F**) Breg subsets in the SLE patients. All *P* values < 0.05, as determined by the Spearman’s rank correlation test.

Based on CD27 and CD38 expression, we further characterized the frequencies of CD27^+^CD38^−^ memory B cells, CD27^+^CD38^+^ plasma B cells, CD27^−^CD38^+^ transitional B cells, and CD27^−^CD38^−^ naive B cells among CD3^−^CD19^+^ B lymphocytes by flow cytometry (Fig. 4A). Subsequent comparisons on the abundance of these distinct B cell subsets between the HCs and SLE patients revealed that the SLE patients had significantly higher frequencies of CD27^+^CD38^−^ memory B cells, CD27^+^CD38^+^ plasma B cells, and CD27^−^CD38^+^ transitional B cells (Fig. 4B). Meanwhile, a significant decrease in the frequency of CD27^−^CD38^−^ naive B cells was observed in the SLE patients (Fig. 4B). Furthermore, we noted that the plasma IL-35 level in the SLE patients was negatively correlated with the frequency of the CD27^+^CD38^−^ memory B cell population (*P* = 0.0020, r = −0.4390; Fig. 5A). Similarly, we observed a negative correlation between the frequency of CD27^+^CD38^−^ memory B cells with the frequencies of IL-35^+^ B cells (*P* = 0.0025, r = −0.4313; Fig. 5B) and IL-10^+^ B cells (*P* = 0.0016, r = −0.4480; Fig. 5C) in the SLE patients. Additionally, the positive correlations between the frequency of CD27^−^CD38^−^ naive B cells and the plasma IL-35 level (*P* = 0.0402, r = 0.3004; Fig. 5D), the frequency of IL-35^+^ B cells (*P* = 0.0438, r = 0.2954; Fig. 5E), and the frequency of IL-10^+^ B cells (*P* = 0.0327, r = 0.3120; Fig. 5F) were observed in the SLE patients.

### The frequencies of Breg subsets and the plasma IL-35 levels were correlated with the clinical indicators in new-onset SLE patients

**Figure 6:**
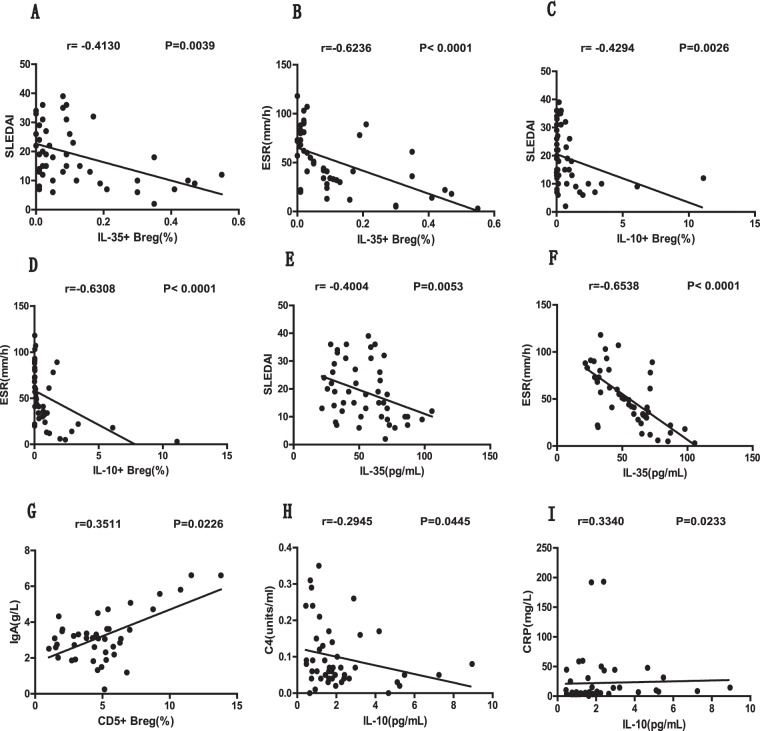
The analyses of correlations among the percentages of different Breg subsets, plasma cytokine levels, and SLE clinical parameters in newly diagnosed SLE patients. **(A**,**B)** The correlation between the percentage of circulating IL-35^+^ Bregs and the SLEDAI score **(A)** or ESR level **(B)**. **(C**,**D)** The correlation between the percentage of circulating IL-10^+^ Bregs and the SLEDAI score **(C)** or ESR level **(D)**. **(E**,**F)** The correlation between the plasma IL-35 level and the SLEDAI score **(E)** or ESR level **(F)**. **(G)** The correlation between the percentage of circulating CD5^+^ Bregs and the plasma IgA level. **(E**,**F)** The correlation between the plasma IL-10 level and the plasma C4 **(E)** or CRP **(F)** level. SLEDAI, SLE disease activity index; ESR, erythrocyte sedimentation rate; CRP, C-reactive protein. All *P* values < 0.05, as determined by the Spearman’s rank correlation test.

**Figure 7:**
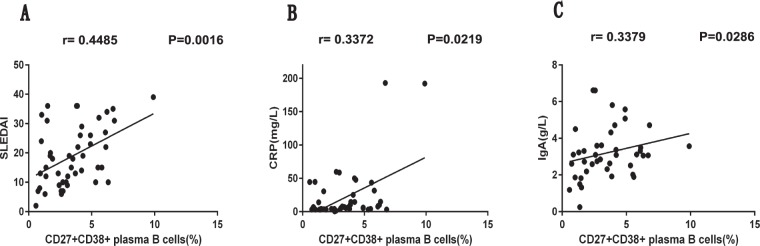
The analyses of correlations between the percentage of the circulating CD27^+^CD38^+^ plasma B cell subset and the SLE clinical parameters in newly diagnosed SLE patients. **(A**–**C)** The correlation between the percentage of circulating CD27^+^CD38^+^ plasma B cells and the SLEDAI score **(A)**, plasma CRP level **(B)**, or plasma IgA level **(C)** in newly diagnosed SLE patients. SLEDAI, SLE disease activity index; CRP, C-reactive protein. All *P* values < 0.05, as determined by the Spearman’s rank correlation test.

To further elucidate the significance of IL-35 expression and B cell suppression in new-onset SLE patients, we evaluated the potential correlations between the frequencies of Breg subsets as well as their secreted cytokine levels and SLE disease severity. Our results demonstrated that the percentages of IL-35^+^ Bregs (*P* = 0.0039, r = −0.4130; Fig. 6A) and IL-10^+^ Bregs (*P* = 0.0026, r = −0.4294; Fig. 6C) as well as the plasma IL-35 levels (*P* = 0.0053, r = −0.4004; Fig. 6E) were inversely correlated with the SLEDAI scores of the SLE patients. Similarly, the ESR also was negatively correlated with the percentages of IL-35^+^ Bregs (*P* < 0.0001, r = −0.6236; Fig. 6B) and IL-10^+^ Bregs (*P* < 0.0001, r = −0.6308; Fig. 6D) as well as the plasma IL-35 levels (*P* < 0.0001, r = −0.6358; Fig. 6F). In addition, we also identified a positive correlation between the frequency of CD5^+^ Bregs and IgA levels (*P* = 0.0226, r = 0.3511; Fig. 6G), a negative correlation between the levels of plasma IL-10 and C4 (*P* = 0.0445, r = −0.2945; Fig. 6H), and a positive correlation between the concentrations of plasma IL-10 and CRP (*P* = 0.0233, r = 0.3340; Fig. 6I). Furthermore, our study also indicated that the frequency of the CD27^+^CD38^+^ plasma B cell subset was positively correlated with the SLEDAI score (*P* = 0.0016, r = 0.4485; Fig. 7A) as well as the concentrations of CRP (*P* = 0.0219, r = 0.3372; Fig. 7B) and IgA (*P* = 0.0286, r = 0.3379; Fig. 7C) in the new-onset SLE patients.

## Discussion

B cells are critical players in the initiation and perpetuation of autoimmunity in SLE^1,2^. Activated B cells not only produce large amounts of autoantibodies, secrete cytokines, and present antigens^14,15^, but they also act as negative regulators of immunity^4,5,7^. In this study, we found that the new-onset SLE patients had higher frequencies of circulating CD27^+^CD38^−^ memory B cells, CD27^+^CD38^+^ plasma B cells, and CD27^−^CD38^+^ transitional B cells than the HCs. The percentage of the plasma B cell subset showed positive correlations with the SLEDAI score and other clinical indicators such as CRP and IgA levels in the SLE patients. Together with previous findings, these observations suggest that the enriched B cell subset may not only play a pathogenic role in the development of SLE but also can be considered as a potential biomarker to assess disease severity. Moreover, we revealed a decreased frequency of CD27^−^CD38^−^ naive B cells in the peripheral blood of SLE patients, which is consistent with previous studies^16^. Furthermore, a similar pattern of changes in the frequencies of different B cell subpopulations was found in the SLE patients. Collectively, these results indicate that imbalanced distributions of different B cell subsets occur in the peripheral blood of patients with newly diagnosed SLE.

In a mouse model of EAE, IL-35^+^ Bregs have been identified to be novel key players in immunosuppression by Shen *et al*.^13^. What’s more, we observed a decreased frequency of blood IL-35^+^ Bregs and a reduced plasma IL-35 concentration in new-onset SLE patients. Notably, the abundance of this subset and its functional cytokines were negatively correlated with the SLEDAI score and ESR value. Our data proposed that IL-35^+^ Bregs and IL-35 might be involved in the pathogenesis of SLE and may act as regulators in autoimmunity. We noted that the percentage of circulating IL-35^+^ Bregs and the plasma IL-35 level were inversely correlated with the levels of the inflammatory cytokines IL-17 and TNF-α, which can be secreted by Th17 cells. Th17 cells are pathogenic and accelerate organ impairment in various animal models and patients with SLE^17–20^. Considering the findings of recent studies indicating the direct suppression of effector T cell proliferation and function by IL-35 *in vitro*^11,21^, we speculated that IL-35^+^ Bregs and their functional cytokine IL-35 might suppress the Th17/IL-17 axis, thus playing a protective role in the pathogenesis of SLE.

Comprising two subunits, IL-12A (p35) and Epstein-Barr virus-induced 3 (EBI3), IL-35 has recently been considered as a new immunosuppressive/anti-inflammatory cytokine^11,22^. IL-35 can mediate signaling either through the heterodimer of receptor chains IL-12R b2/gp130 or the homodimer of each chain^23^. IL-35 is expressed by both Bregs and regulatory T cells (Tregs)^10,24^. In our study, we observed that the IL-35 level was positively correlated with the percentages of circulating IL-35^+^ Bregs and IL-10^+^ Bregs in patients with SLE. These observations indicated that IL-35 was not only produced by the IL-35^+^ Breg subset, but it also might be expressed by IL-10^+^ Bregs, which is consistent with a previous study suggesting that IL-35^+^ Bregs and IL-10^+^ Bregs are overlapping cell subsets^25^. Moreover, we identified a negative correlation between IL-35 expression and the abundance of CD27^+^CD38^+^ plasma cells. Previous studies also have established that IL-35 can expand the numbers of IL-35^+^ Bregs and IL-10^+^ Bregs as well as induce IL-10 production^12,25^, which, combined with our findings, led us to hypothesize that IL-35 may function as a pivotal regulatory cytokine to promote the generation of Bregs by inducing naive B cells to develop into IL-35^+^ Bregs and IL-10^+^ Bregs as well as inhibiting the conversion of pathogenic plasma B cells in the pathogenesis of SLE. We found that the percentage of blood IL-10^+^ Bregs was decreased in the SLE patients. This subset showed a negative correlation with SLE disease severity indicators, including the SLEDAI score and the ESR. Consistent with published reports^6,8,9^, our data also indicated that IL-10^+^ Bregs might possess regulatory and protective properties in SLE progression and that their dynamic changes might be associated with the progression of human SLE.

IL-10 production is the most-studied mechanism of IL-10^+^ Bregs^7,26^, which confers the ability of these cells to maintain tolerance to self-antigens and to suppress inflammation and autoimmune responses^25^. Interestingly, we observed elevated circulating IL-10 levels in the SLE patients. The increased IL-10 level showed no correlations with IL-10^+^ Bregs or other Breg subsets, but it did correlate with SLE disease severity parameters, including C4 (negatively correlated) and CRP (positively correlated), suggesting that the total amount of circulating IL-10 plays a proinflammatory role in SLE. IL-10 is derived from different cell types and exerts dual functions under different contexts in the pathogenesis of autoimmune diseases^27^. Apart from its anti-inflammatory and immunosuppressive effects, IL-10 is also a potent cofactor for B cell survival, proliferation, differentiation, and Ig secretion^28^. Besides Breg-derived IL-10, IL-10 is produced by monocytes and T cells in SLE patients^29–31^. Together with previous studies^6,8,9,32^, we speculate that IL-10^+^ B cell-derived IL-10 comprises only a small fraction of the pool of IL-10 in patients with SLE. In a mouse model of lupus, Kalampokis *et al*. identified that IL-10^+^ Bregs regulate autoimmune responses and have protective and potentially therapeutic effects^31^. However, besides the fact that IL-35 can be produced by IL-10^+^ Bregs as discussed above, other mechanisms of action of IL-10-independent Breg cell subsets, including the production of IgM, the generation of adenosine, and the expression of programmed death-ligand 1 or Fas ligand, still need to be clarified in future studies.

CD5 has been shown to be expressed on a subpopulation of B cells with unique properties^3^. CD5^+^ Bregs can produce pathogenic autoantibodies and present antigens to T cells. Alternatively, they can also secrete IL-10^7^. Thus, the CD5^+^ Breg subset is identified as an important regulator of autoimmunity and an inducer of immune tolerance^3^. Our study demonstrated a reduced frequency of circulating CD5^+^ Bregs in patients with newly diagnosed SLE, which was positively correlated with the IgA level. However, no correlation was found between the frequency of blood CD5^+^ Bregs and the plasma IL-10 level. CD5^+^ Bregs are more likely to be involved in SLE by the dynamic changes of their number and the pathogenic antibodies they produce. Since a reduced number of CD5^+^ Bregs may correspondingly result in the reduced production of IL-10 by CD5^+^ Bregs, IL-10 from other sources might play a predominant role in the pathogenesis of SLE.

In conclusion, our study suggests that IL-35 expression is reduced in patients with newly diagnosed SLE, as both the frequency of circulating IL-35^+^ Bregs and the level of plasma IL-35 are significantly decreased in new-onset SLE patients. Our work only focused on Breg subsets and their roles in SLE pathogenesis, and the involvement of other regulatory cell subsets like Tregs, plasmacytoid dendritic cells (pDCs) or myeloid-derived suppressor cells (MDSCs) cannot be ignored. They also play very important roles in the pathogenesis of SLE. Tregs can suppress the activation, expansion, and differentiation of multiple types of cells including CD4+ T helper cells, CD8+ T cells, and B cells^33–35^. And some studies have already reported reduced numbers or impaired function of circulating Tregs in SLE patients, though some others found no apparent abnormalities and even increased levels of Tregs in SLE as compared with healthy controls^36^. As to pDCs, an aberrant regulatory feedback was found between pDCs and Bregs in SLE^37^. There were studies showed that MDSCs significantly suppressed the proliferation of CD4+ T cells, and the production of IFN-γ was significantly decreased in CD4+ T cells co-cultured with MDSCs^38^. Although a further study with a larger sample size and multiple time points is required to decipher the molecular and cellular mechanisms of IL-35^+^ Bregs on SLE progression, our current study implies that IL-35^+^ Bregs are important negative autoimmune regulators in SLE initiation and progression, our current study implies that IL-35^+^ Bregs are important negative autoimmune regulators in SLE initiation and progression.

## Materials and Methods

### Study subjects

This study involved a total of 47 new-onset SLE patients who were recruited between November 2016 and January 2018 from the Department of Rheumatology, The First Hospital of Jilin University, Changchun, China. Twenty healthy volunteers, as controls, with matched ethnicity, age, and gender, were recruited from the Physical Examination Center of the Outpatient Department of The First Hospital of Jilin University. These SLE patients were diagnosed according to the revised SLE classification criteria established by the American College of Rheumatology^39^, and they demonstrated clinical symptoms for <3 months without receiving any treatments. The disease activity of these new-onset SLE patients was assessed by the SLEDAI^40^, and active disease was indicated by an SLEDAI score >6. A total of 20 HCs, with matched age, gender, and ethnicity, were enrolled from the Physical Examination Center of Jilin University, Changchun, China. The participants with any kind of other autoimmune disorder, malignancy, or microbial infection, or those who had been treated with immunosuppressive reagents within the past 6 months were excluded from this study. All the experimental procedures involving human samples were conducted with strict adherence to the guidelines of the Declaration of Helsinki. The study protocol was approved by the Human Ethics Committee of Jilin University, and a written informed consent was obtained from each subject.

### Clinical information and laboratory testing

The clinical information, including the demographic and clinical characteristics of all of the participants, was acquired from the general hospital records. The routine laboratory tests, including the assays for complete blood cell counts and measuring the plasma levels of anti-dsDNA antibodies, anti-Sm antibodies, IgG, IgA, IgM, C3, C4, ESR, and CRP, were performed as described previously^41^.

### Isolation of human peripheral blood mononuclear cells (PBMCs)

After overnight fasting, each participant was subjected to peripheral blood drawing (6 mL/person), and the PBMCs were isolated by density-gradient centrifugation with Ficoll-Paque PLUS density gradient media (GE Healthcare Bio-Sciences, Pittsburgh, PA, USA). All plasma samples were immediately distributed into tubes and stored at −80 °C for later analysis. PBMCs at a concentration of 1 × 10^6^/mL were maintained in Roswell Park Memorial Institute (RPMI)-1640 culture medium supplemented with 10% fetal bovine serum (FBS; Thermo Fisher Scientific, Waltham, MA, USA) and stored at 4 °C for subsequent flow cytometric analysis.

### Flow cytometry

The staining buffer used for flow cytometry was Hank’s Balanced Saline Solution without calcium or magnesium and supplemented with 2% heat-inactivated FBS.

PBMCs resuspended in the staining buffer were used for the surface staining of CD3, CD19, CD27, and CD38 as well as the subsequent intracellular staining of IL-10, EBI3, and p35. For surface staining, the cells were incubated with the indicated antibodies for 30 min at 4 °C. For intracellular staining, the cells were first stimulated in duplicate with 50 ng/ml phorbol myristate acetate (PMA; Sigma-Aldrich, St. Louis, MO, USA), 1.0 µg/ml ionomycin(Sigma-Aldrich) in the presence or absence of 50 ng/ml lipopolysaccharide (LPS; Sigma-Aldrich) for 2 h, and then cultured for another 4 h in the presence of monensin (2 µl, GolgiStop; BD Biosciences, San Jose, CA, USA). After surface staining for 30 min, the cells were fixed and permeabilized with the Fixation/Permeabilization Solution Kit (BD Biosciences), according to the manufacturer’s protocols. The following antibodies were used in this study: FITC-anti-CD3 (BioLegend, San Diego, CA, USA), PerCP/Cy5.5-anti-CD5 (BioLegend), APC-H7-anti-CD19, BV510-anti-CD27, PE-Cy7-anti-CD38, BV421-anti-IL-10, PE-anti-EBI3, and eFluor® 660-anti-p35 (all unspecified antibodies were from BD Biosciences; FITC, fluorescein isothiocyanate; PE, Phycoerythrin; PerCP, Peridinin Chlorophyll Protein; APC, allophycocyanin; BV, brilliant violet). Control staining was performed with the following isotype-matched control antibodies: FITC-anti-mouse IgG2a, PerCP/Cy5.5-anti-mouse IgG1, APC-H7-anti-mouse IgG1, BV510-anti-mouse IgG1, PE-Cy7-anti-mouse IgG1, BV421-anti-mouse IgG1, PE-anti-mouse IgG2b, and eFluor® 660-anti-mouse IgG1 (all from BD Biosciences). All flow cytometric analyses were performed using a FACSAria II instrument (BD Biosciences), and data were analyzed by FlowJo software (version 7.6.2; FlowJo, LLC, Ashland, OR, USA).

### Enzyme-linked immunosorbent assay (ELISA)

The plasma IL-35 level was measured using a human IL-35 ELISA kit (Thermo Fisher Scientific), according to the manufacturer’s instructions. The detection limit of this kit was 15.6 pg/mL, and the IL-35 levels were determined by referring to the standard curve.

### Quantitation of plasma cytokine levels by cytometric bead array (CBA)

The levels of plasma IL-10, IL-17A, TNF-α, and IFN-γ were measured using the CBA method, as described previously^42^. A FACSAria II flow cytometer was used for data acquisition. The kits were purchased from BD Biosciences, and the experimental procedures as well as data analysis were performed according to the manufacturer’s instructions.

### Statistical analysis

Data were analyzed using the Statistical Package for the Social Sciences software (version 21.0; IBM, Armonk, NY, USA). Quantitative data were represented as the mean ± standard error of the mean, with each dot in the plots denoting an individual value. The Kruskal-Wallis analysis of variance test followed by the Dunn-Bonferroni post hoc method or the Mann-Whitney U test was used to evaluate differences between the indicated groups. The Spearman’s rank correlation test was employed to analyze correlations between the indicated groups. *P* values < 0.05 were considered statistically significant.
